# A Case of Focal Segmental Glomerulosclerosis With Immune Complexes: Is HIV, Hepatitis B, or Crack the Culprit?

**DOI:** 10.7759/cureus.17335

**Published:** 2021-08-20

**Authors:** Patil Balozian, Abdul Rahman Al Armashi, Mohammad Haidous, Massiel Cruz-Peralta, Keyvan Ravakhah

**Affiliations:** 1 Internal Medicine, St. Vincent Charity Medical Center, Cleveland, USA

**Keywords:** hiv associated nephropathy (hivan), hiv-associated immune complex kidney disease (hivick), non collapsing focal segmental glomerulosclerosis (fsgs), polyclonal gammopathy, light chains, nephropathy, proteinuria, immune-complex kidney disease, acute tubular necrosis (atn), human immunodeficiency virus-1(hiv-1)

## Abstract

Human immunodeficiency virus (HIV)-positive individuals are at an increased risk for kidney diseases, including HIV-associated nephropathy (HIVAN), focal segmental glomerulosclerosis (FSGS), HIV immune complex disease of the kidney (HIVICK), and acute tubular necrosis (ATN). Non-modifiable factors such as age and genetics, as well as modifiable factors such as illicit drug use and compliance, define the progression to renal failure. The patient is a 64-year-old African American male with HIV, treated latent syphilis, chronic kidney disease stage 3a, and cocaine use disorder who presented with shortness of breath, bilateral lower extremities swelling, and fatigue with normal vitals and a physical exam remarkable for bibasilar inspiratory crackles with peripheral edema. Laboratory tests showed creatinine (Cr) of 2.23 mg/dL with a baseline of 1.5 mg/dL, albumin of 1.8, blood natriuretic peptide (BNP) of 667.88, and lipidemia. His urine was remarkable for proteinuria and microalbuminuria in the presence of cocaine. Immunofixation electrophoresis showed a marked increase in IgG and IgM, free lambda, and free kappa/free lambda ratio with HIV viral load of 39,400 copies/ml, absolute CD4 count of 56, and an acute hepatitis B panel. Renal biopsy confirmed HIVAN with FSGS accompanied by collapsing features, HIVICK, and ATN. The patient was subsequently started on highly active antiretroviral therapy (HAART) with prophylactic antibiotics and close monitoring.

## Introduction

Human immunodeficiency virus type 1 (HIV-1) seropositive patients are at an increased risk for kidney diseases, including HIV-associated nephropathy (HIVAN), focal segmental glomerulosclerosis (FSGS), HIV immune complex disease of the kidney (HIVICK), and acute tubular necrosis (ATN). Nonmodifiable factors such as age and genetics, as well as modifiable factors such as illicit drug use and compliance, define the progression to renal failure. The most common cause of chronic renal failure in HIV-1-seropositive patients is HIVAN, almost exclusively seen in black patients. It was primarily described in 1984 by Rao et al. and Pardo et al. in New York and Miami and is currently a definite clinical and histopathologic entity [1-3]. In this article, we present the case history of a 64-year-old African American male with HIV who presented with nephropathy and was found to have HIVAN, HIVICK, and ATN. 

## Case presentation

The patient is a 64-year-old African American man with HIV, treated latent syphilis, chronic kidney disease stage 3a, and cocaine use disorder who presented with progressive, exertional shortness of breath, orthopnea, paroxysmal nocturnal dyspnea, bilateral lower extremities swelling, and fatigue of a couple of weeks duration. Review of systems was unremarkable. He reported non-compliance to HIV medications for the past three years (diagnosed 17 years ago) and had no scheduled medications. The patient was vitally stable with a physical exam remarkable for bibasilar inspiratory crackles and +2 pitting edema on lower extremities. Laboratory tests (Table 1) showed normal hemoglobin, creatinine (Cr) of 2.23 mg/dL with a baseline of 1.5 mg/dL, blood urea nitrogen (BUN) of 27 with a glomerular filtration rate of 36 mL/min, albumin of 1.8 g/dl, blood natriuretic peptide (BNP) of 667.88 ng/ml, and lipidemia. His urine was remarkable for proteinuria with a microalbumin/creatinine ratio of 3364.2 mcg/mg in the presence of cocaine. Immunofixation electrophoresis showed a marked increase in IgG and IgM with a mild increase in free kappa, free lambda, and free kappa/free lambda ratio. Antineutrophil cytoplasmic antibodies (ANCA), myeloperoxidase antibody, proteinase 3 (PR3) antibody, C3, and C4 were normal. HIV viral load was 39,400 copies/ml with an absolute CD4 count of 56. Hepatitis B surface antigen and hepatitis B e antigen were positive. Echocardiography showed diastolic heart failure with preserved ejection fraction and the patient was started on intravenous furosemide for symptomatic relief. 

**Table 1:** Laboratory Work BUN: blood urea nitrogen, LDL: low-density lipoprotein, ANCA: anti-neutrophil cytoplasmic antibodies, PCR: polymerase chain reaction

Preliminary Laboratory work	Value	Reference range
Hemoglobin	13.7 g/dl	14.0-16.5 g/dl
Creatinine	2.23 g/dl	0.70-1.30 g/dl
BUN	27mg/dl	7-18 mg/dl
Albumin	1.8 g/dl	2.9-4.4 g/dl
Cholesterol	218 mg/dl	<200 mg/dl
LDL Cholesterol	141 mg/dl	60-130 mg/dl
BNP	667.88 ng/ml	0.01-0.045 ng/ml
Urine Studies	Value	Reference range
White Blood Count	11-15/ High Power Field	0-5/ High Power Field
Urine Protein	100mg/dl	Negative
Random Creatinine	29.10 mg/dl	30-115 mg/dl
Random Microalbumin	979.0 mg/dl	<30 mg/dl
Creatinine	29.1 mg/dl	30-115 mg/dl
Microalbumin/Creatinine	3364.2 mcg/mg	0-30 mcg/mg CR
Cocaine Screen	Positive	Negative
Immunology	Value	Reference range
Immunofluorescence IgG	4085 mg/dl	603-1613 mg/dl
Immunofluorescence IgA	216 mg/dl	61-437 mg/dl
Immunofluorescence IgM	469 mg/dl	20-172 mg/dl
Anti-Proteinase 3	<3.5 U/ml	0-3.5 U/ml
Atypical p-ANCA	<1:20 titer	<1:20 titer
p-ANCA antibody	<1:20 titer	<1:20 titer
Myeloperoxidase antibody	<9 U/ml	0-9 U/ml
ANCA	1:40 titer	<1:20 titer
Complement C3	112 mg/dl	90-180 mg/dl
Complement C4	19.2 mg/dl	10-40 mg/dl
CD4 Cells (% percentage)	6 (% percentage)	30-61 (% percentage)
Absolute CD4 count	56 cells/ul	490-1740 cells/ul
Free Kappa Light Chain	351.8 mg/L	3.3-19.4 mg/L
Free Lambda Light Chain	167.5 mg/L	5.7-26.3 mg/L
Free Kappa/Lambda Ratio	2.10	0.26-1.65
Serology	Value	Reference Range
Hepatitis Bs Antigen	Positive	Negative
Hepatitis Be Antigen	Positive	Negative
Hepatitis Be Antibody	Negative	Negative
HIV RNA copies/ml Ultra	39400	20-10,000,000 copies/ml
HIV-1 RNA (PCR) log 10	4.595	Log10 copy/ml

**Figure 1:**
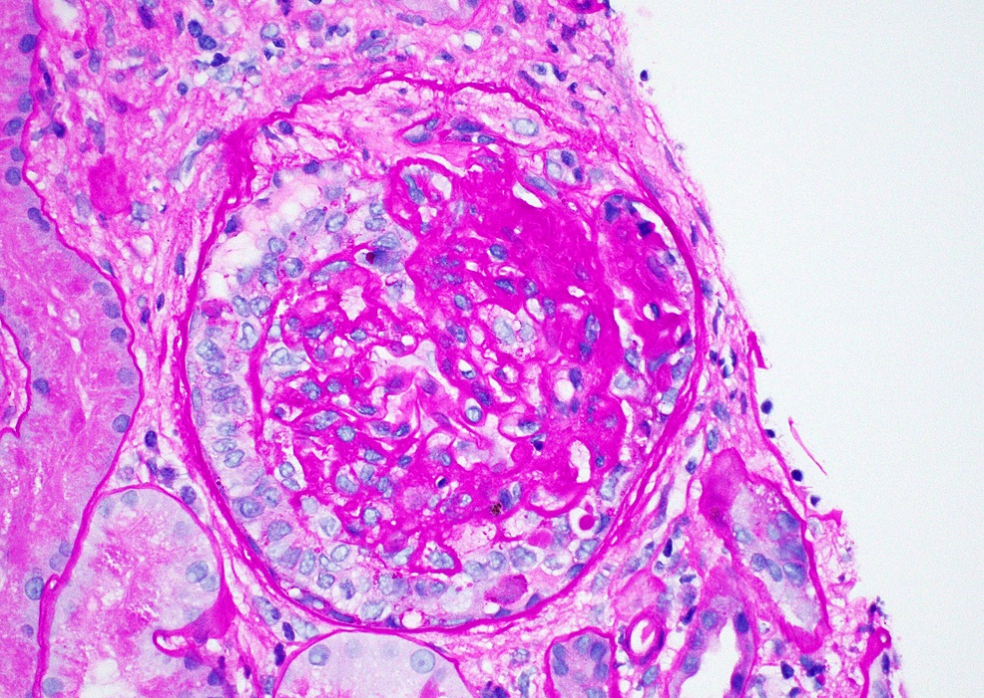
Renal Biopsy Renal biopsy histology demonstrated segmental sclerosis with crescentic features indicative of focal segmental glomerulosclerosis.

**Figure 2:**
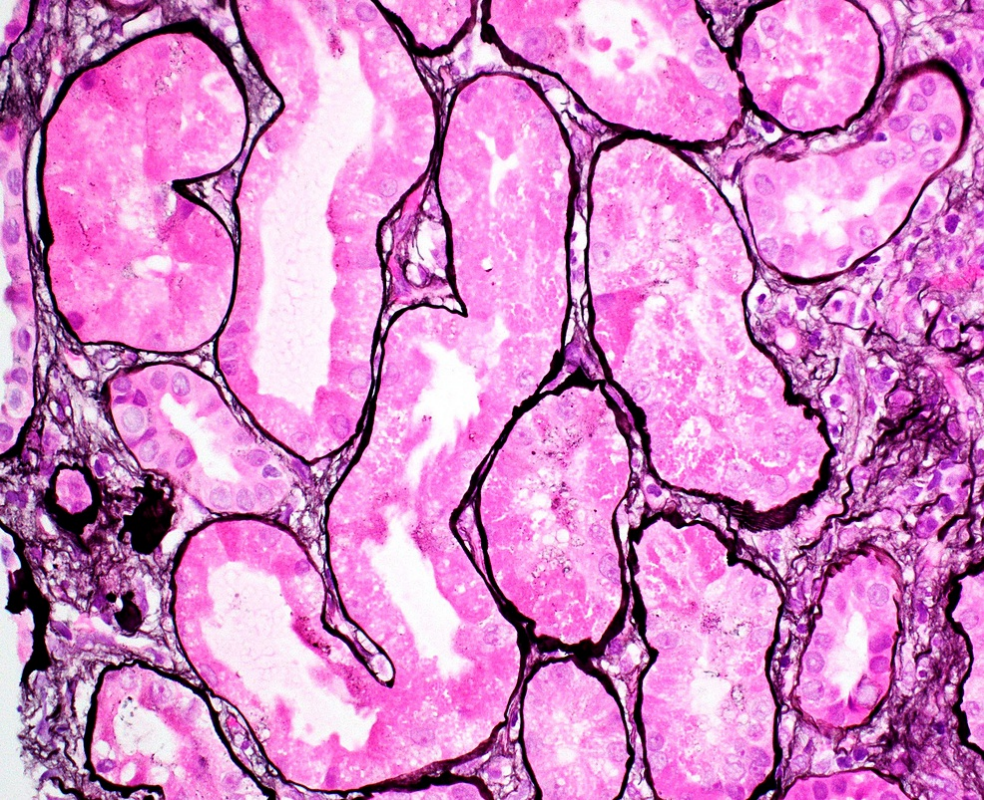
Renal Biopsy Renal biopsy histology demonstrated denudation of renal tubular cells and loss of brush borders.

Renal ultrasound showed increased parenchymal echogenicity in both kidneys followed by a right renal biopsy that revealed FSGS with collapsing features, acute tubular injury, and mild to moderate interstitial fibrosis. There were 17 glomeruli, two of which were completely sclerotic with findings of focal and mild mesangial hypercellularity. Up to five glomeruli had features of focal segmental glomerulosclerosis, including bowman’s capsular adhesions, segmentally solidified capillary lumina, and urinary space collagen (Figure 1). In addition, there was mild to moderate mononuclear cell inflammation noted in the interstitium with non-atrophic tubules showing features of injury such as apical cytoplasmic blebbing, broken brush borders, and tubular cell mitotic figures (Figure 2). 

**Figure 3:**
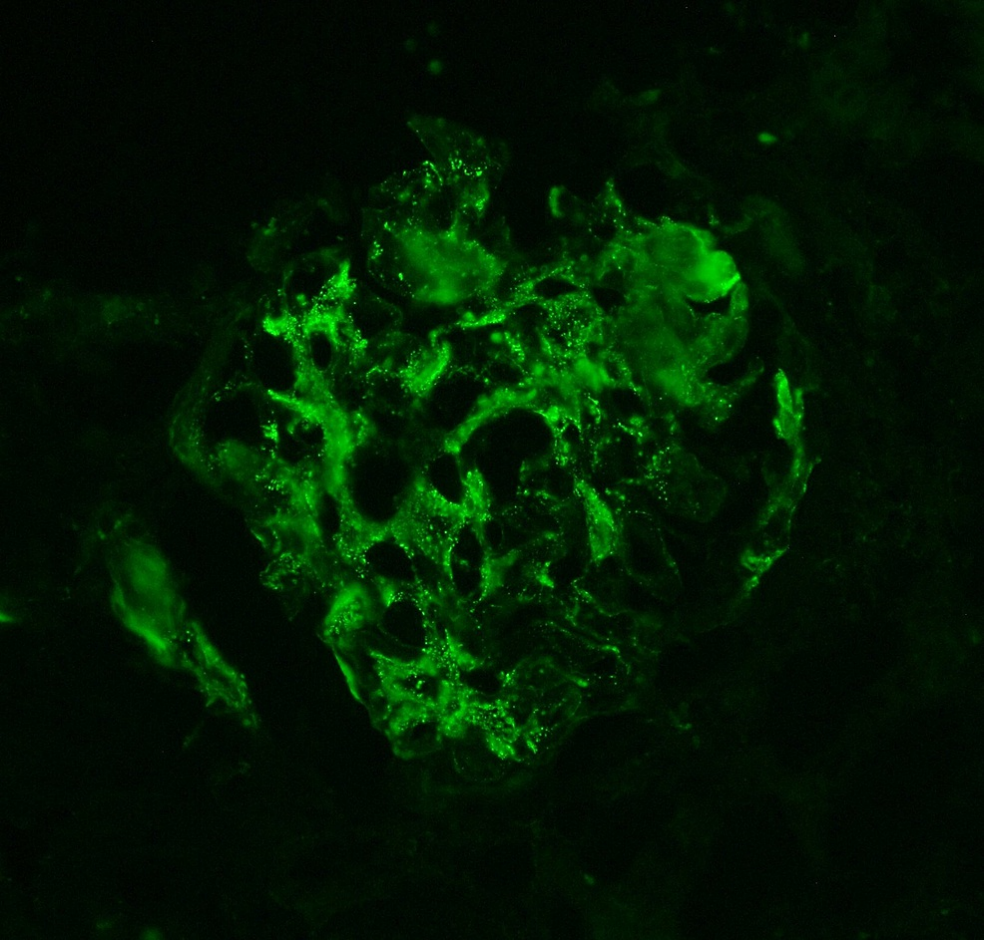
Immunofluorescence Microscopy Immunofluorescence microscopy demonstrated mesangial regions stained for IgA (1+), IgM (3+), C3 (trace), kappa (trace to 1+), and lambda (trace to 1+) in a granular pattern.

Immunofluorescence microscopy consisted of mesangial regions and capillary walls staining mostly for IgA and IgM. Three out of 11 glomeruli were completely sclerotic with mesangial regions and capillary walls staining for IgA (1+), IgM (3+), C3 (trace), kappa (trace to 1+), and lambda (trace to 1+) in a granular pattern (Figure 3). Tubular epithelial cell protein reabsorption droplets were stained for albumin (3+), kappa (1 to 2+), and lambda (1 to 2+).

**Figure 4:**
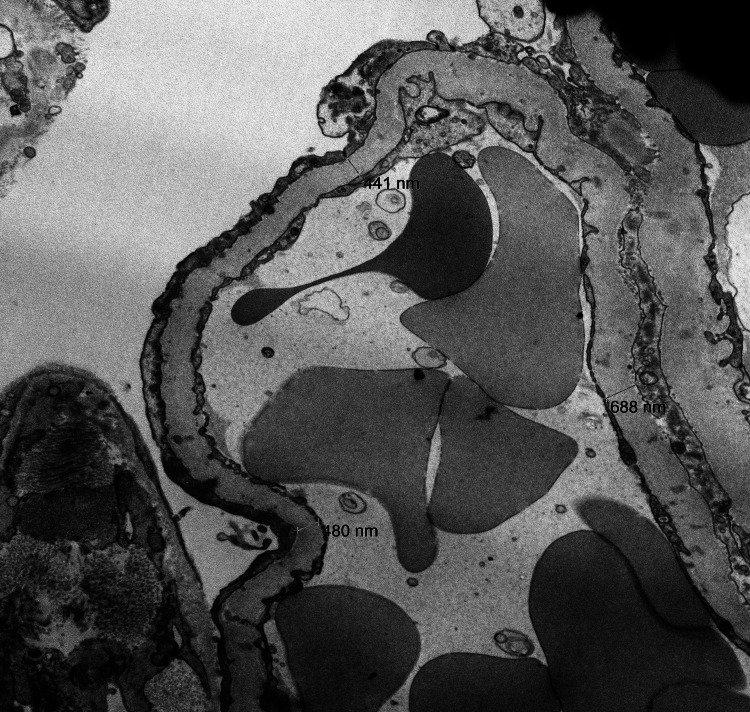
Electron Microscopy Electron microscopy demonstrated effacement of podocyte foot processes with electron dense deposits in the mesangial, subendothelial, subepithelial and intramembranous regions.

Electron microscopy demonstrated extensive effacement of podocyte foot processes. Electron dense deposits were located in mesangial regions with few in the subendothelial, subepithelial, and intramembranous regions (Figure 4). Glomerular basement membrane had the normal trilaminar structure with moderate thickening. 

The patient was observed to have HIVAN and FSGS with collapsing features, HIVICK, and ATN. He improved symptomatically throughout his hospitalization, was discharged on highly active antiretroviral therapy (HAART) with prophylactic antibiotics, and was recommended to follow up very closely with nephrology and infectious disease specialists. 

## Discussion

HIVAN is a distinct clinicopathologic syndrome predominantly involving African American patients. Susceptibly to genetic mutations of APOL1 in patients of African descent is associated with the development of FSGS [4]. As per the literature, patients with HIVAN are mostly black with CD4 <200/mL (P = 0.01) and glomerular filtration rate <30 mL/min/1.73m^2^ (P < 0.01) [5]. HIVAN’s overall prevalence varies according to the population's demographic features with ∼10% to 15% of HIV-infected patients developing HIVAN [1]. 

HIVAN can occur at any stage of the HIV infection, although most patients show significant immunosuppression and advanced infection at the time of the diagnosis. Its histopathologic features are represented in about 80% of cases by focal segmental glomerulosclerosis with collapse of the glomerular tuft in some glomeruli without prominent mesangial expansion or basement membrane thickening, extensive tubular ectasia, and tubulointerstitial changes [1]. 

The occurrence of hepatitis B, being a well-known cause of membranous nephropathy (MN), has also been reported in FSGS [6]. There have been previous cases, seven reported, two of which demonstrated hepatitis B surface antigen in the renal tissue and a response to lamivudine, indicating a possible causal association between the viral infection and occurrence of nephrotic syndrome [7]. As to the patient’s cocaine use disorder, it is likely the contributing etiology of his ATN [8]. Also, his panel depicts a polyclonal gammopathy that is not uncommonly reported in patients with HIV infection. Polyclonal hypergammaglobulinemia in turn promotes the development of circulating immune complexes, their passive trapping, or the in situ deposition of the antibodies binding to HIV viral antigens [9].

In terms of pathogenesis, HIV nucleic acid in podocytes, parietal epithelial cells, tubular epithelial cells, T-cells, and macrophages in human HIVAN renal biopsy specimens support the presence of the HIV gene. The kidney acts as a compartment separate from the blood where HIV-1 can replicate even in patients with serological remission [10].

Clinically, the classic presentation of HIVAN includes rapidly progressive renal failure, moderate to nephrotic range proteinuria, bland urinary sediment, and ultrasound findings of large, highly echogenic kidneys [11]. Studies regarding the optimal treatment of HIVAN with HIVICK patients involve initiation of HAART, steroids, and angiotensin-converting enzyme inhibitors [12]. In multivariate analysis, HIVAN risk was reduced by 60% (95% CI, 30 to 80%) by use of HAART, and no patient developed HIVAN when HAART had been initiated prior to the development of AIDS [13]. Also, the pathogenic role of HIV replication in the development of HIVICK for patients on HAART reveals improvement of kidney function in patients who have detectable HIV RNA at the time that HIVICK diagnosis has been studied [14].

## Conclusions

This case highlights that HIV-positive patients are at an increased risk of developing complex focal glomerular, immune, and tubular kidney pathologies, especially in the setting of acute infections, drugs, and non-compliance. HIVAN and HIVICK can coexist in some cases, mostly in the context of patients being off HAART with low CD4 counts and high viral loads. Hepatitis B could cause FSGS in a small percentage of patients, less likely in our patient’s case. Cocaine use disorder contributes to ATN. Nephrologists and infectious disease specialists should work together to screen a seropositive population with important proteinuria (>1 g/24 h) consistent with HIVAN and confirm the diagnosis by renal biopsy. Although therapies for this condition have produced contradicting results, HAART may prolong nephropathic patient survival. Keen follow-up of proteinuria and kidney function remains vital.
